# Spatial analysis improves the detection of early corneal nerve fiber loss in patients with recently diagnosed type 2 diabetes

**DOI:** 10.1371/journal.pone.0173832

**Published:** 2017-03-15

**Authors:** Dan Ziegler, Karsten Winter, Alexander Strom, Andrey Zhivov, Stephan Allgeier, Nikolaos Papanas, Iris Ziegler, Jutta Brüggemann, Bernd Ringel, Sabine Peschel, Bernd Köhler, Oliver Stachs, Rudolf F. Guthoff, Michael Roden, German Diabetes Study (GDS) Group

**Affiliations:** 1 Institute for Clinical Diabetology, German Diabetes Center at Heinrich Heine University, Leibniz Center for Diabetes Research, Düsseldorf, Germany; 2 Department of Endocrinology and Diabetology, Medical Faculty, Heinrich Heine University, Düsseldorf, Germany; 3 German Center for Diabetes Research (DZD), München-Neuherberg, Germany; 4 Institute of Anatomy, University of Leipzig, Leipzig, Germany; 5 Department of Ophthalmology, University of Rostock, Rostock, Germany; 6 Institute for Applied Computer Science, Karlsruhe Institute of Technology, Karlsruhe, Germany; Hirosaki Daigaku, JAPAN

## Abstract

Corneal confocal microscopy (CCM) has revealed reduced corneal nerve fiber (CNF) length and density (CNFL, CNFD) in patients with diabetes, but the spatial pattern of CNF loss has not been studied. We aimed to determine whether spatial analysis of the distribution of corneal nerve branching points (CNBPs) may contribute to improving the detection of early CNF loss. We hypothesized that early CNF decline follows a clustered rather than random distribution pattern of CNBPs. CCM, nerve conduction studies (NCS), and quantitative sensory testing (QST) were performed in a cross-sectional study including 86 patients recently diagnosed with type 2 diabetes and 47 control subjects. In addition to CNFL, CNFD, and branch density (CNBD), CNBPs were analyzed using spatial point pattern analysis (SPPA) including 10 indices and functional statistics. Compared to controls, patients with diabetes showed lower CNBP density and higher nearest neighbor distances, and all SPPA parameters indicated increased clustering of CNBPs (all P<0.05). SPPA parameters were abnormally increased >97.5^th^ percentile of controls in up to 23.5% of patients. When combining an individual SPPA parameter with CNFL, ≥1 of 2 indices were >99^th^ or <1^st^ percentile of controls in 28.6% of patients compared to 2.1% of controls, while for the conventional CNFL/CNFD/CNBD combination the corresponding rates were 16.3% vs 2.1%. SPPA parameters correlated with CNFL and several NCS and QST indices in the controls (all P<0.001), whereas in patients with diabetes these correlations were markedly weaker or lost. In conclusion, SPPA reveals increased clustering of early CNF loss and substantially improves its detection when combined with a conventional CCM measure in patients with recently diagnosed type 2 diabetes.

## Introduction

Diabetic sensorimotor polyneuropathy (DSPN) which affects around one-third of all diabetic patients [1] predicts diabetic foot ulcers [2], cardiovascular morbidity [3], and mortality [4]. Thus, objective measures to accurately detect early nerve pathology indicating incipient DSPN that may be more susceptible to intervention than late-stage alterations are required. One such an emerging non-invasive technique, corneal confocal microscopy (CCM), is being used to assess the corneal subbasal nerve plexus (SNP) localized between the basal epithelium and Bowman’s membrane to quantify corneal nerve fiber pathology in patients with or without clinically manifest DSPN [5,6]. Most widely used and generally accepted corneal nerve parameters include corneal nerve fiber length (CNFL), density (CNFD), and branch density (CNBD) [7]. Using these measures, numerous studies have demonstrated that corneal nerve fiber loss augments in relation to increasing severity of DSPN [5,6] and also may precede the development of clinical DSPN [8].

It is generally agreed that it is important to understand ways to optimize the ability of CCM to serve as a sensitive and specific marker of DSPN [9]. A worldwide normative reference database has recently been established to provide the basis for wider use of CCM as a diagnostic test [7]. Indeed, recent studies indicate continuing efforts aimed at improving the diagnostic performance of CCM, albeit with limited success. Different corneal regions such as the corneal apex (central cornea) and the whorl-like area usually located 1–2 mm inferior to the corneal apex have been compared, but yielded contrasting results with respect to diagnostic accuracy [10,11]. Standardizing CNFL for nerve tortuosity resulted only in a marginal improvement in diagnostic performance compared to CNFL [9]. Furthermore, the vast majority of previous studies using CCM in diabetes patients have analyzed relatively small image frames of 0.16 mm^2^ that may not be representative of larger corneal areas. As possible solutions, both multiple non-overlapping image frames per patient or larger mosaic images generated from image sequences have been proposed [12–14].

The aforementioned conventional CCM measures adequately reflect SNP morphometry, but cannot describe the spatial configuration of corneal nerve fiber networks. This can be theoretically accomplished using methods that characterize spatial point patterns on different scales and estimate the presence of a spatial dependence among the points [15,16]. These methods enable the distinction between random, regular or clumped patterns. In this comprehensive study, we analyzed the spatial distribution of branching points from skeletonized subbasal corneal nerve fiber networks both on nearest neighbor level and over larger distances. We hypothesized that the early corneal nerve fiber loss in recent-onset type 2 diabetes subjects reported using conventional CCM morphometry [14] follows a clustered rather than random distribution pattern which could contribute to improving the detection of early SNP abnormalities shortly after diabetes diagnosis.

## Materials and methods

The study was conducted in accordance with the Declaration of Helsinki and was approved by the ethics committee of Heinrich Heine University, Düsseldorf, Germany. All participants provided a written informed consent. Included were 86 patients with recently diagnosed type 2 diabetes and 47 age- and sex-matched controls. Patients with diabetes were participants of the German Diabetes Study (GDS), which evaluates the long-term course of diabetes and its sequelae (ClinicalTrials.gov Identifier: NCT01055093) [17]. Inclusion criteria for entry into the GDS are type 1 or type 2 diabetes, known diabetes duration ≤1 year and age of 18–69 years at baseline assessment. Exclusion criteria for the present study were type 3 diabetes, pregnancy, severe diseases (cancer), psychiatric disorders, immunosuppressive therapy, limited cooperation ability, corneal disorders, and neuropathy from causes other than diabetes. Inclusion criteria for the control group were age of ≥18 years and normal OGTT [18], while exclusion criteria were neuropathy from any cause and those applied to the diabetes group. Among type 2 diabetes patients from the GDS who were asked to participate in the present study, approximately 50% agreed. Control subjects were volunteers largely recruited by newspaper and online advertisement.

### CCM examination

CCM was performed using a Heidelberg Retina Tomograph II (HRT II) with the Rostock Cornea Module-RCM (Heidelberg Engineering, Heidelberg, Germany) as previously described [14]. In brief, the acquired images had a resolution of 384 x 384 pixels and the field of view was 0.16 mm^2^. Experienced ophthalmologists (AZ and SP), who were blinded to all study data, except for CCM, carried out the examinations. Using a modified, oscillating volume scan operating mode of the HRT II, in which the focus plane of the microscope is continually shifted back and forth, a number of image stacks (with an axial image distance of 0.5 μm) were acquired for each patient. The stack size was individually adjusted to the height of present ridge-like tissue deformations [14,19,20]. A stack size of 96 images (scan depth: 48 μm) was chosen for ridge heights of less than 48 μm, and 120 images (60 μm) otherwise. At least three scans were performed and one or more mosaic images of the SNP generated for each patient [21,22]. The total duration of microscopy was about 15 minutes. The following conventional CCM parameters were determined as previously reported [14]: corneal nerve fiber length (CNFL), defined as the total length of all nerve fibers (mm/mm^2^); corneal nerve fiber density (CNFD), defined as the number of nerve fiber segments per mm^2^; and corneal nerve branch density (CNBD), defined as the number of branching points per mm^2^.

Ocular surface examination including lid margins, cornea and conjunctiva was performed to exclude eyes of controls and patients with dry eye signs and symptoms. Subjects wearing contact lenses were excluded, while in those with history of e.g. corneal scars or inflammation in one eye, the non-affected eye was examined.

### Spatial point pattern analysis

The coordinates of branching points in thinned fiber network images and the respective binary masks of the valid image areas from the segmentation images (actual acquired SNP layer content) formed the basis for the automated quantitative spatial analysis. A more detailed description is available in the Supporting Information (S1 Supporting Information).

For each image the number of branching points (BPs) and point density (BPD) (number of BPs normalized to the acquired image area) were determined. Empty space distance (ESD) between BPs was calculated by applying a distance transform to the whole image area. The resulting distance map characterizes the empty space extents between BPs and contains distance information of all image pixels to the nearest BP. All local distance map values were averaged to calculate the mean ESD (cardinality of empty space) of an image. Spatially adjacent BPs were determined using a Delaunay triangulation [23] algorithm that was modified for application on image areas with irregular border shape. Nearest neighbor indices were derived from distances between neighboring BPs from which the minimum (MINN) and mean (MENN) nearest neighbor distances along with their respective standard deviations (MINNSD and MENNSD) were calculated. MINNSD and MENNSD provide information on the homogeneity or fluctuation of distances between adjacent BPs. The mean Voronoi cell area (VCA) with its standard deviation (VCASD) of the point patterns was computed as an alternative measure of inter-point distance based on Voronoi tesselation [24]. VCA reflects the amount of empty space around single BPs while VCASD describes its variation. The spatial structure of point patterns (random, regular or clustered) on a nearest neighbor level was characterized by the edge-corrected Clark-Evans aggregation index (CEAI) [25,26].

Each spatial point pattern was then submitted to second-order spatial analysis which provided information about the spatial structure of point patterns over different scales. This analysis included the calculation of Besag's L-function [27] which is the standardized and easier to interpret version of Ripley's K-function [28] and the pair-correlation-function [29] as well as the visualization of these functions along with their respective Monte-Carlo envelopes [28]. Tests for complete spatial randomness (CSR) of point patterns based on L- and pair-correlation (L, PC) functions were performed using the Maximum Absolute Deviation (MAD) test [28,30] that provided the test statistics for deviation from CSR (MADL and MADPC, respectively). This test indicates the presence and the degree of clustering within a given point pattern. To test for differences between spatial point patterns of the control and diabetic groups as a whole, the studentized permutation test [31] for L-functions and pair-correlation functions was employed and the latter were visualized along with their Monte-Carlo envelopes in the two groups. This test allows the direct comparison of point pattern groups on the basis of their functional summary characteristics.

### Peripheral nerve function

Peripheral nerve function tests were performed as previously described [14]. Motor NCV was measured in the median, ulnar, and peroneal nerves, while sensory NCV and sensory nerve action potentials (SNAP) were determined in the median, ulnar, and sural nerves at a skin temperature of 33–34°C using surface electrodes (Nicolet VikingQuest, Natus Medical, San Carlos, CA). Quantitative sensory testing included measurement of the vibration perception threshold (VPT) on the medial malleolus using the method of limits (Vibrameter, Somedic, Stockholm, Sweden) and thermal detection thresholds (TDT) including warm and cold thresholds on the dorsum of the foot using the method of limits (TSA-II NeuroSensory Analyzer, Medoc, Ramat Yishai, Israel). Neurological examination was carried out using the Neuropathy Disability Score (NDS) and Neuropathy Symptom Score (NSS) [32]. These and all other clinical examinations were performed by operators, who were blinded to the corneal findings in all subjects.

### Statistical analysis

Continuous data were expressed as mean±SD. Categorical data were given as absolute or relative frequencies with 95% CI and were analyzed by Fisher's exact test. For normally distributed data, parametric tests (t-test or Pearson product-moment correlation), otherwise nonparametric tests (Mann-Whitney U test or Spearman rank correlation) were applied. To determine associations between two variables, univariate correlations and multiple linear regression analyses were performed. The level of significance was set at α = 0.05.

## Results

The demographic and clinical characteristics of the patients and controls have been published elsewhere [14]. The mean number of corneal nerve BP was 142.83±98.80 in the controls and 88.65±70.32 in the diabetes group (P<0.001), while BPD was 0.00024±0.00011 n/pixel in the controls and 0.00018±0.00010 n/pixel in the diabetic subjects (P<0.001). The mean values of SPPA parameters are shown in Table 1. Nine of the 10 indices studied were significantly higher, while CEAI was lower in the diabetes group as compared to the control subject (all P<0.05).

**Table 1:** Parameters of spatial analysis of corneal nerve fibers in the diabetic and control groups studied.

	Control (n = 47)	Diabetes (n = 86)	*P* value
ESD (μm)	47.57 ± 15.93	69.21 ± 46.59	<0.001
MINN (μm)	26.65 ± 6.80	31.20 ± 11.07	0.013
MINNSD (μm)	22.83 ± 6.74	28.86 ± 13.88	0.019
MENN (μm)	75.70 ± 26.56	85.92 ± 32.98	0.011
MENNSD (μm)	56.56 ± 21.41	67.46 ± 27.98	0.006
VCA (μm^2^)	5287 ± 4034	7994 ± 7080	0.005
VCASD (μm^2^)	4673 ± 2661	8535 ± 9724	0.002
CEAI	0.6821 ± 0.1034	0.6244 ± 0.1362	0.028
MADL	28.93 ± 14.31	40.45 ± 26.92	0.029
MADPC	38.08 ± 27.44	60.43 ± 57.64	0.017

Values are mean±SD. MINN: minimum nearest neighbor distances between branching points, MENN: mean nearest neighbor distances between branching points, MINNSD: standard deviation of minimum nearest neighbor distances between branching points, MENNSD: standard deviation of mean nearest neighbor distances between branching points, CEAI: Clark and Evans aggregation index, ESD: mean empty space distance for a branching point pattern, VCA: Voronoi cell area, VCASD: standard deviation of Voronoi cell area, MADL: maximum absolute deviation from complete spatial randomness based on L-functions, MADPC: maximum absolute deviation from complete spatial randomness based on pair-correlation functions, CSR: complete spatial randomness

Table 2 shows the percentages of abnormal SPPA parameters above the 97.5^th^ percentile and abnormal CEAI and CNFL below the 2.5^th^ percentile of the control group. The percentages of abnormal MINN, MINNSD, VCASD, MADL, and CNFL were significantly higher in the diabetes group compared to the control subjects (all P<0.05), while borderline significance was observed for MADPC (P = 0.051). No significant differences between the groups were noted for the rates of abnormal MENN, MENNSD, CEAI, ESD, and VCA. The most favorable discriminatory power was observed for MADL, MINN, and MINNSD. The highest rate of abnormal values in the diabetes group was found for MADL (23.5%), while MINN showed the lowest percentage of abnormal values among controls (2.1%).

**Table 2:** Percentages (95% CIs) of subjects with abnormal spatial point pattern analysis parameters >97.5^th^ percentile and abnormal CEAI and CNFL *<2.5^th^ percentile of the control group.

	Control (n = 47)	Diabetes (n = 86)	*P* value
ESD (%)	8.5 (3.0–18.4)	18.6 (12.0–26.9)	0.136
MINN (%)	2.1 (0.1–9.7)	19.3 (12.5–27.8)^†^	0.005
MINNSD (%)	4.3 (0.8–12.8)	21.7 (14.5–30.4)^†^	0.01
MENN (%)	4.3 (0.8–12.8)	8.4 (4.0–15.3)^†^	0.487
MENNSD (%)	6.4 (1.8–15.7)	12.0 (6.7–19.6)^†^	0.374
VCA (%)	4.3 (0.8–12.8)	14.5 (8.6–22.4)^†^	0.084
VCASD (%)	6.4 (1.8–15.7)	21.7 (14.5–30.4)^†^	0.026
CEAI (%)*	4.3 (0.8–12.8)	11.9 (6.6–19.4)^‡^	0.210
MADL (%)	4.3 (0.8–12.8)	23.5 (16.2–32.3)^§^	0.003
MADPC (%)	4.3 (0.8–12.8)	16.5 (10.2–24.5)^§^	0.051
CNFL (%)*^||^	4.3 (0.8–12.8)	18.6 (12.0–26.9)	0.019

^||^Previously published in ref. [14];

missing values:

^§^n = 1;

^‡^n = 2;

^†^n = 3.

MINN: minimum nearest neighbor distances between branching points, MENN: mean nearest neighbor distances between branching points, MINNSD: standard deviation of minimum nearest neighbor distances between branching points, MENNSD: standard deviation of mean nearest neighbor distances between branching points, CEAI: Clark and Evans aggregation index, ESD: mean empty space distance for a branching point pattern, VCA: Voronoi cell area, VCASD: standard deviation of Voronoi cell area, MADL: maximum absolute deviation from complete spatial randomness based on L-functions, MADPC: maximum absolute deviation from complete spatial randomness based on pair-correlation functions, CSR: complete spatial randomness, CNFL: corneal nerve fiber length

Table 3 displays the percentages of combined abnormalities in ≥1 out of 2 and ≥1 out of 3 SPPA parameters above 97.5^th^ and 99^th^ percentiles and corneal nerve fiber length (CNFL) below 2.5^th^ and 1^st^ percentiles of the control group, respectively. Among the four combinations including two parameters, one of which was CNFL, three showed inadequately high rates of ≥1 abnormality in the controls (8.5%) when using the <2.5^th^/>97.5^th^ percentiles, while the <1^st^/>99^th^ percentiles yielded an appropriate range in the controls (2.1–6.4%) and highest percentage for the CNFL/MADL combination (31.8%) which was markedly higher than that obtained with the individual SPPA measures or CNFL. The combination of two SPPA indices showed the highest rate of ≥1 abnormal parameter for MINNSD/MADL >97.5^th^ percentile of 35.3% in the diabetes group compared to 6.4% in controls. When combining three parameters, one of which was CNFL, the best discriminatory power between diabetic and control subjects was obtained using the combination CNFL <1^st^ percentile/MINN/MADL >99^th^ percentile with 38.8 vs 6.4% showing ≥1 abnormal parameter. In contrast, the combination of ≥1 abnormality out of 3 conventional parameters (CNFL, CNFD, CNBD) showed percentages identical with those obtained with CNFL as a single parameter (18.6 vs 4.3%).

**Table 3:** Prevalence (95% CIs) of combined abnormal (≥1 out of 2 and ≥1 out of 3) parameters of Spatial Point Pattern Analysis (SPPA) above 97.5^th^ and 99^th^ percentiles and CNFL, CNFD, aor CNBD below 2.5^th^ and 1^st^ percentiles of control group.

	Control (n = 47)	Diabetes (n = 86)	*P* value
*CNFL and SPPA parameter combined*			
CNFL <2.5^th^ / MINN >97^th^ centile (%)	4.3 (0.8–12.8)	29.8 (21.6–39.0)	<0.001
CNFL <1^st^ / MINN >99^th^ centile (%)	2.1 (0.1–9.7)	28.6 (20.5–37.8)	<0.001
CNFL <2.5^th^ / MINNSD >97^th^ centile (%)	8.5 (3.0–18.4)	34.5 (25.9–44.0)	0.001
CNFL <1^st^ / MINNSD >99^th^ centile (%)	6.4 (1.8–15.7)	27.4 (19.5–36.5)	0.003
CNFL <2.5^th^ / MADL >97^th^ centile (%)	8.5 (3.0–18.4)	35.3 (26.7–44.7)	0.001
CNFL <1^st^ / MADL >99^th^ centile (%)	6.4 (1.8–15.7)	31.8 (23.5–41.1)	0.001
CNFL <2.5^th^ / VCASD >97^th^ centile (%)	8.5 (3.0–18.4)	33.3 (24.8–42.7)	0.001
CNFL <1^st^ / VCASD >99^th^ centile (%)	4.3 (0.8–12.8)	31.0 (22.7–40.3)	<0.001
*Two SPPA parameters combined*			
MINN / MADL >97^th^ centile (%)	6.4 (1.8–15.7)	34.1 (25.6–43.5)	<0.001
MINN / MADL >99^th^ centile (%)	6.4 (1.8–15.7)	30.6 (22.4–39.8)	0.001
MINNSD / MADL >97^th^ centile (%)	6.4 (1.8–15.7)	35.3 (26.7–44.7)	<0.001
MINNSD / MADL >99^th^ centile (%)	6.4 (1.8–15.7)	25.9 (18.2–34.9)	0.006
MADL / VCASD >97^th^ centile (%)	8.5 (3.0–18.4)	29.4 (21.3–38.6)	0.004
MADL / VCASD >99^th^ centile (%)	4.3 (0.8–12.8)	25.9 (18.2–34.9)	0.002
*CNFL and two SPPA parameters combined*			
CNFL <2.5^th^ / MINN / MADL >97^th^ centile (%)	8.5 (3.0–18.4)	41.2 (32.2–50.7)	<0.001
CNFL <1^st^ / MINN / MADL >99^th^ centile (%)	6.4 (1.8–15.7)	38.8 (29.9–48.3)	<0.001
CNFL <2.5^th^ / MINNSD / MADL >97^th^ centile (%)	10.6 (4.3–21.1)	43.5 (34.4–53.0)	<0.001
CNFL <1^st^ / MINNSD / MADL >99^th^ centile (%)	8.5 (3.0–18.4)	35.3 (26.7–44.7)	0.001
CNFL <2.5^th^ / MADL / VCASD >97^th^ centile (%)	10.6 (4.3–21.1)	38.8 (29.9–48.3)	0.001
CNFL <1^st^ / MADL / VCASD >99^th^ centile (%)	6.4 (1.8–15.7)	35.3 (26.7–44.7)	<0.001
CNFL / CNFD / CNBD <2.5^th^ centile (%)	4.3 (0.8–12.8)	18.6 (12.0–26.9)	0.019
CNFL / CNFD / CNBD <1^st^ centile (%)	2.1 (0.1–9.7)	16.3 (10.1–24.3)	0.011

MINN: minimum nearest neighbor distances between branching points, MINNSD: standard deviation of minimum nearest neighbor distances between branching points, VCASD: standard deviation of Voronoi cell area, MADL: maximum absolute deviation from complete spatial randomness based on L-functions, CNFL: corneal nerve fiber length, CNFD: corneal nerve fiber density, CNBD: corneal nerve branch density

The correlations between CNFL and SPPA parameters are shown in Table 4. Associations were highly significant for all indices in the control group, whereas in the diabetes group the correlation coefficients were markedly lower, and no significant associations with CNFL were noted for MINNSD, MENNSD, CEAI, and MADPC.

**Table 4:** Correlations between Corneal Nerve Fiber Length (CNFL) and parameters of spatial point pattern analysis.

	Control (n = 47)		Diabetes (n = 86)	
	*r*	*P* value	*r*	*P* value
BP	0.767	<0.001	0.332	0.002
BPD	0.925	<0.001	0.402	<0.001
ESD	-0.909	<0.001	-0.365	0.001
MINN	-0.688	<0.001	-0.244	0.026
MINNSD	-0.765	<0.001	-0.118	0.290
MENN	-0.804	<0.001	-0.259	0.018
MENNSD	-0.844	<0.001	-0.169	0.127
VCA	-0.788	<0.001	-0.374	0.001
VCASD	-0.869	<0.001	-0.275	0.012
CEAI	0.658	<0.001	0.197	0.072
MADL	-0.654	<0.001	-0.220	0.043
MADPC	-0.693	<0.001	-0.110	0.318

BP: number of branching points, BPD: branching point density, MINN: minimum nearest neighbor distances between branching points, MENN: mean nearest neighbor distances between branching points, MINNSD: standard deviation of minimum nearest neighbor distances between branching points, MENNSD: standard deviation of mean nearest neighbor distances between branching points, CEAI: Clark and Evans aggregation index, ESD: mean empty space distance for a branching point pattern, VCA: Voronoi cell area, VCASD: standard deviation of Voronoi cell area, MADL: maximum absolute deviation from complete spatial randomness based on L-functions, MADPC: maximum absolute deviation from complete spatial randomness based on pair-correlation functions

**Fig 1:**
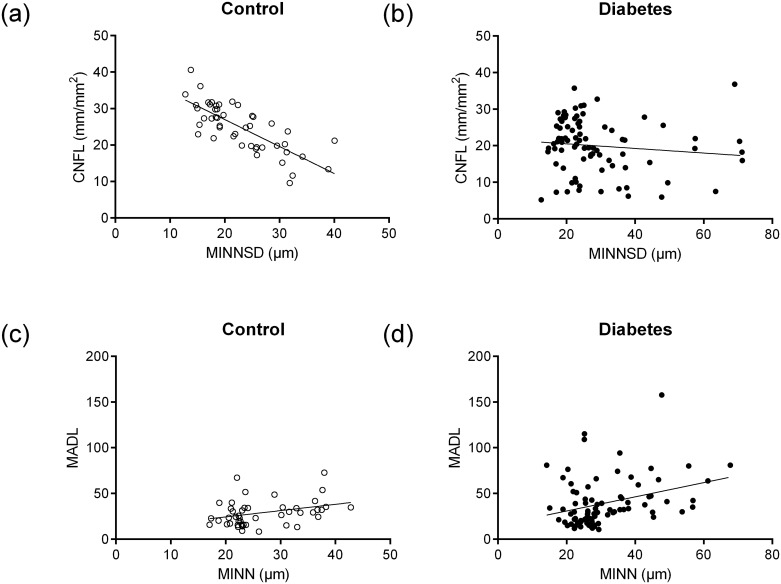
A, B. Correlations between CNFL and MINNSD in control (r = -0.765; P<0.001) and diabetic subjects (r = -0.118; P = 0.290). C, D: Correlations between MADL and MINN in control (r = 0.321; P = 0.028) and diabetic subjects (r = 0.320; P = 0.003).

Fig 1A and 1B illustrates the high correlation between CNFL and MINNSD in the control group (r = -0.765; P<0.001) and lack of correlation between these indices in the diabetes group (r = -0.118; P = 0.290). Fig 1C and 1D illustrates the relatively weak correlations between MADL and MINN in the control group (r = 0.321; P = 0.028) and diabetes group (r = 0.320; P = 0.003).

The highest correlations of SPPA parameters with age and measures of peripheral nerve function in the control group were found for MENNSD, CEAI, ESD, MADL, and MADPC (Table 5). In the diabetes group, these associations were considerably weaker and rarely statistically significant (data not shown).

**Table 5:** Correlations between selected parameters of spatial analysis and age and measures of peripheral nerve function in the control group.

	ESD	MENNSD	CEAI	MADL	MADPC
Age	0.294*	0.289	-0.467*	0.306*	0.511*
Median motor NCV	-0.344*	-0.312*	0.357*	-0.251	-0.308*
Peroneal motor NCV	-0.122	-0.144	0.394*	-0.223	-0.340*
Ulnar sensory NCV	-0-248	-0-165	0.442*	-0.330*	-0.383*
Sural SNAP	-0.388*	-0.367*	0.409*	-0.340*	-0.425*
Metacarpal VPT	0.467*	0.482*	-0.453*	0.445*	0.536*
Malleolar VPT	0.341*	0.383*	-0.493*	0.337*	0.515*
Warm TDT foot	0.397*	0.314*	-0.319*	0.330*	0.392*
Cold TDT foot	-0.283	-0.191	0.393*	-0.211	-0.402*

*P<0.05.

NCV: nerve conduction velocity, SNAP: sensory nerve action potential, VPT: vibration perception threshold, TDT: thermal detection threshold, MENNSD: standard deviation of mean nearest neighbor distances between branching points, CEAI: Clark and Evans aggregation index, ESD: mean empty space distance for a branching point pattern, MADL: maximum absolute deviation from complete spatial randomness based on L-functions, MADPC: maximum absolute deviation from complete spatial randomness based on pair-correlation functions

The correlations between the SPPA parameters in the entire study population are shown in Table 6. Each of these indices correlated significantly with one another, except for CEAI and MINN. The highest correlation coefficients (r>0.8) were obtained between MINN, MENN, VCA and their SDs as well as between VCA, VCASD, MENN, and MENNSD. The lowest correlation coefficients (r<0.35) were found for the relationships between CEAI and MINN, MINNSD, and MENN as well as between MINN and both MADL and MADPC.

**Table 6:** Correlation coefficients (r) for the relationships between parameters of spatial analysis in the entire study population (n = 133).

	MINN	MINNSD	MENN	MENNSD	CEAI	ESD	VCA	VCASD	MADL
MINNSD	0.869*								
MENN	0.858*	0.838*							
MENNSD	0.654*	0.758*	0.887*						
CE	-0.009	-0.197*	-0.330*	-0.531*					
ESD	0.600*	0.714*	0.782*	0.866*	-0.698*				
VCA	0.761*	0.793*	0.908*	0.854*	-0.504*	0.928*			
VCASD	0.644*	0.759*	0.807*	0.870*	-0.603*	0.977*	0.944*		
MADL	0.341*	0.531*	0.499*	0.713*	-0.704*	0.862*	0.692*	0.837*	
MADPC	0.309*	0.537*	0.559*	0.678*	-0.745*	0.781*	0.705*	0.753*	0.726*

*P<0.05.

MINN: minimum nearest neighbor distances between branching points, MENN: mean nearest neighbor distances between branching points, MINNSD: standard deviation of minimum nearest neighbor distances between branching points, MENNSD: standard deviation of mean nearest neighbor distances between branching points, CEAI: Clark and Evans aggregation index, ESD: mean empty space distance for a branching point pattern, VCA: Voronoi cell area, VCASD: standard deviation of Voronoi cell area, MADL: maximum absolute deviation from complete spatial randomness based on L-functions, MADPC: maximum absolute deviation from complete spatial randomness based on pair-correlation functions

**Fig 2:**
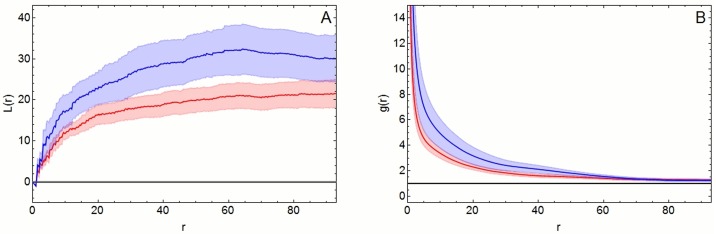
A, B. Pooled L-functions (A) and pair-correlation functions (B). Both functions show a higher deviation from complete spatial randomness (CSR) point patterns (blue line) in diabetic patients than in controls (red line). The respective Monte-Carlo envelopes (blue and red areas) in A are separated for the most part, while they are slightly superimposed in B. Results from studentized permutation test confirm these visual findings. A significant difference between the groups was noted for L functions (p = 0.026) but not for pair-correlation functions (p = 0.056).

The studentized permutation test showed a significant difference in the spatial point patterns between controls and diabetic patients for L functions (p = 0.026), but not pair-correlation functions (p = 0.056). The pooled graphs showed a higher deviation from CSR in point patterns of diabetes patients than those of controls (Fig 2).

## Discussion

In this study using a novel approach of SPPA we demonstrate increased clustering of corneal nerve branching points (CNBPs) indicating a patchy pattern of early corneal nerve fiber loss in recently diagnosed type 2 diabetes patients. Among the 10 individual SPPA parameters studied, the highest discriminatory power was obtained for MADL, which was abnormally increased in 23.5% of diabetes patients and 4.3% of controls. Using a conservative percentile approach and the definition of ≥1 of 3 indices being abnormal, the conventional CNFL/CNFD/CNBD combination yielded abnormality in 16.3% of the diabetes subjects and in 2.1% of the controls, whereas the corresponding rates increased to 28.6 vs 2.1% when combining MINN with CNFL. Thus, the combination of an individual SPPA parameter with a conventional CCM measure achieved a substantially increased detection of early pathology indicating not only CNF loss but also its spatial pattern of enhanced clustering, while the specificity in controls remained identical. The highest rate of abnormality (38.8%) was achieved when combining MINN and MADL with CNFL, albeit this approach came at the cost of a concomitant increase in the controls (6.4%), thus indicating lower specificity.

In early studies, even cases with mild DSPN showed loss of myelinated fibers, a decrease in the median diameter, and an increase in the variability of density among frames and among fascicles that began in proximal nerve and extended to distal levels. These findings were explained by multifocal fiber loss along the length of nerves and sprouting [33]. Likewise, intraepidermal nerve fiber (IENF) loss due to neuropathy does not seem to occur in a random way, but rather, the remaining nerves seem arranged in ‘clusters’ and exhibit some spatial pattern, possibly due to collateral branching by the surviving nerve fibers [34,35]. Preliminary attempts to describe this pattern indicate that the spatial distribution of nerve fibers in the foot becomes more ‘clustered’ as neuropathy advances [35].

Previous CCM studies indicated a variable nature of corneal nerve fiber loss in advanced neuropathy, but quantitative analyses have not been undertaken [36,37]. Although the relationship between the right and left corneal nerve fiber morphometry was highly significant among control subjects and diabetic patients with increasing severity of neuropathy, this was not the case in patients with severe neuropathy. It has been suggested that this may reflect variability and perhaps the patchy nature of central corneal nerve damage in advanced neuropathy, which has been also shown in a small whole corneal nerve mapping study in a diabetic patient with severe neuropathy [36,37]. Qualitative comparison of the two maps showed an overall reduction in the number of nerve fibers in the diabetic patient with neuropathy. The density of the entire nerve plexus was reduced and branching was markedly reduced. Overall, there seemed to be fewer nerve fibers in the inferior and temporal sectors and the inferior whorl seems to be affected more severely [37]. Since the spatial distribution of the SNP fiber networks shows considerable variability across the cornea, the relatively small size of the widely used corneal area (0.16 mm^2^) may contribute to variable results of the conventional CCM parameters [38].

This is the first study addressing the spatial pattern of CNBP loss. We reported early CNF loss with percentages of CNFL <2.5^th^ percentile of 18.6% in the present group of recent-onset type 2 diabetes patients [14]. Here we extend this finding by demonstrating that SPPA has the potential to markedly improve the diagnostic performance of CCM in detecting early corneal nerve fiber loss in diabetes by adding data on the non-random distribution pattern of CNBPs to conventional CCM morphometry.

The properties of the various SPPA indices used herein and their different discriminatory performance deserve comment. While BPs only reflect the total number of CNBPs in an image, BPD represents a weighted measure due to its dependence on the actual size of the image area and correlates better with CNFL which is calculated in the same context. MINN and MENN are both calculated from adjacent CNBPs. While MINN measures the distance to the single nearest CNBP only, MENN averages the distances to all adjacent CNBPs. Higher SDs of these two parameters reveal irregular distance patterns caused by inhomogeneous spatial distribution of CNBPs. In this study, both MINN and MINNSD were clearly more sensitive indices in detecting increased early clustering in diabetic patients than MENN and MENNSD.

The empty space between CNBPs is being characterized by both ESD and VCA. ESD is calculated using a distance transformation for all pixels of the image area [39]. VCA is based on a tiling of the image plane, where the “area of influence” of each CNBP is computed and represented by a corresponding Voronoi cell [40]. Hence, these two methods handle empty space from fundamentally different perspectives. ESD correlated better with CNFL than VCA in controls in terms of empty space characterization, but VCASD can be used to characterize the underlying spatial point pattern regarding irregular distances. We found similarly high rates of abnormalities in the diabetes group for VCASD and MINNSD which can both be used in combination with CNFL to improve the diagnostic performance of CCM.

Among the nearest neighbor indices, CEAI can practically be used for the distinction of the three basic point patterns. A randomly distributed pattern shows CEAI = 1.0, regularity of a point pattern is indicated by CEAI>1.0, while a clumped or clustered point pattern can be assumed for CEAI<1.0 [25]. This parameter reveals that both controls and diabetes subjects show rather clustered patterns, but it also indicates that patterns are more clustered in those with diabetes. However, CEAI was relatively insensitive in discriminating between controls and diabetes subjects and thus, may not be useful in detecting early increased clustering in diabetic patients in clinical practice. Since CEAI is calculated only from the number of CNBPs, image area, and image border, this parameter does not consider the SD of neighboring distances which obviously detects early clustering of corneal nerve fiber loss more sensitively.

Furthermore, we employed second-order analysis that characterizes point patterns on a far greater scale. L functions and pair-correlation functions display the spatial configuration of the analyzed point patterns (random, regular or clustered) along with the respective radius *r* at which these features occur. The L function includes all points within the radius *r*, while the pair-correlation function only involves points between the radius *r* and a narrow annulus of diameter *d*. Thus, the results of the L function at larger distances are always influenced by the shorter distances within the point patterns. This may obscure the spatial association at any given scale.

In order to quantify the departure from CSR, L functions and pair-correlation functions were tested for CSR using the Maximum Absolute Deviation test. The resulting test statistic provides a measure for this deviation, and the results showed that MADPC was slightly more sensitive than MADL in discriminating between point patterns between control and diabetes subjects. Better applicability of non-cumulative pair-correlation functions and considerably lower reliability of cumulative L functions was already reported for the analysis of IENFs [35].

L functions and pair-correlation functions can be computed for point patterns of individual images as well as for groups of images. Such pooled graphs provide additional information since they show the average spatial configuration at group level. The pooled spatial patterns can also be tested for group differences using the studentized permutation test. This test allows the direct comparison of point pattern groups on the basis of their functional summary characteristics without the need for extracting scalar characteristics from the functions and thereby omitting valuable spatial information. In contrast to the slightly better performance of the pair-correlation function in conjunction with MADPC, the studentized permutation test yields better results for the L function. This apparent contradiction arises from the two different approaches. MADL and MADPC are based on the deviation from CSR, while the studentized permutation test directly compares the two-point pattern groups and investigates group differences without regard to CSR [31]. Nevertheless, departure from CSR and the distinctly higher degree of clustering in diabetic subjects can be derived from the pooled graphs of the L and pair correlation functions. In practical terms, the studentized permutation test yielded results compatible with the categorical approach of classifying the rates of abnormally increased clustering which gave preference to MADL over MADPC. Thus, we selected MADL for combination with CNFL to optimize the diagnostic performance of CCM.

Interesting observations were the strong correlations between CNFL and SPPA measures in the control group as opposed to the diabetes group in which these correlations were considerably weaker or even lacking. It is conceivable that these parameters are associated only if CNBP clustering is within the normal range. In contrast, significant clustering may not be adequately reflected by reduced CNFL. The weak correlation of SPPA measures with CNFL in the diabetes group underlines the usefulness of SPPA as an additional CCM measure largely independent of the conventional ones.

A major strength of this work is the inclusion of a comprehensive array of SPPA parameters using state of the art methods of functional statistics applied to a relatively large and homogenous study population with recently diagnosed type 2 diabetes. A limitation is the relatively small control sample and the cross-sectional nature of this study so that the predictive value and further course of the described increased spatial clustering cannot be determined at present. Finally, selection bias cannot be excluded, since patients and controls included in this study may not be representative of the general population.

## Conclusions

The present study suggests that the detection of early corneal nerve fiber loss in recent-onset type 2 diabetes patients is considerably fostered by SPPA as a novel technique to analyze CNBPs added to conventional CCM morphometry. This approach yields a markedly increased detection of early pathology with a high rate of reduced CNFL and/or increased clustering corneal nerve fiber loss in 28.6% of diabetes patients compared with 2.1% of controls. The precise temporal sequence of enhanced clustering in relation to the incidence, severity, and progression of DSPN remains to be established in prospective studies.

## Supporting information

S1 Supporting Information Detailed description of spatial point pattern analysis. (DOCX)
