# Supplementary material for: Spatial analysis improves the detection of early corneal nerve fiber loss in patients with recently diagnosed type 2 diabetes
Source: PLoS One. 2017 Mar 15;12(3):e0173832. doi: 10.1371/journal.pone.0173832 (PMC5352008; doi:10.1371/journal.pone.0173832)
Supplement: S1 Supporting Information — (DOCX) [file pone.0173832.s001.docx]

**Ziegler et al., Spatial analysis improves the detection of early corneal nerve fiber loss in recently diagnosed type 2 diabetes**

**Supporting Information**

**Methods**

**Spatial point pattern analysis (SPPA)**

**Summary of SPPA**

The coordinates of branching points in thinned fiber network images and the respective binary masks of the valid image areas from the segmentation images (actual acquired SNP layer content) formed the basis for the automated quantitative SPPA which was performed in a masked fashion by one of us (KW).

For each image the number of branching points (BPs) and point density (BPD) (number of points divided by binary mask area) were determined. The number of BPs reflects the actual number of nerve branches within the acquired image area and BPD is the normalized version of BPs in relation to the acquired image area. Dense nerve fiber networks possess higher numbers of BPs and a higher BPD, while a low number of BPs / lower BPD indicate scarce or heavily interrupted networks. It reflects a weighted measure that can be used for the comparison of images with widely differing areas. For every image pixel a local value was calculated based on the distance to the nearest BP and all local values were subsequently averaged to calculate the empty space distance (ESD: µm) of an image. This parameter represents the cardinality of empty space between points. Dense BP patterns contain narrow empty areas, while sparse patterns may express vast connected empty areas indicating poorly connected nerve fiber networks.

Spatially adjacent BPs were determined using a Delaunay triangulation algorithm that was modified for application on image areas with irregular border shape. Nearest neighbor indices were derived from distances between neighboring BPs from which the minimum (MINN) and mean (MENN) nearest neighbor distances along with their respective standard deviations (MINNSD and MENNSD) were calculated. MINNSD and MENNSD provide information about homogeneity or fluctuation of distances between adjacent BPs. The mean Voronoi cell area (VCA: µm²) with its standard deviation (VCASD) of the point patterns was computed as an alternative measure of inter-point distance based on Voronoi tesselation. VCA indicates how much empty area can be found around the individual BPs. The BP cells consist of all space that is closer to their respective BP than to any other. Dense BP patterns contain a high number of small area cells, while sparse patterns may express fewer cells with larger areas, the latter indicating poorly connected nerve fiber networks. VCASD describes the variation of Voronoi cell sizes and provides further information on the homogeneity or fluctuation of distances between adjacent BPs.

The spatial structure of point patterns (random, regular or clustered) on a nearest neighbor level was characterized by the edge-corrected Clark-Evans aggregation index (CEAI) which measures the degree, to which a point pattern differs from random distribution. A randomly distributed pattern (homogeneously configured nerve fiber network) shows CE=1.0, regularity of a point pattern is indicated with CE>1.0, while a clumped or clustered point pattern (clumped or clustered nerve fiber network) can be assumed when CE<1.0. With this dimensionless parameter, the spatial structure of point patterns can be directly characterized and different point patterns can be compared to each other.

To analyze the spatial structure of point patterns for clustering, their departure from complete spatial randomness (CSR) was investigated, whereby CSR acts as a ”dividing line“ between regular and clustered patterns. Each spatial point pattern was then submitted to second-order spatial analysis which included the calculation of Besag's L-function which is the standardized and easier to interpret version of Ripley's K-function and the pair-correlation-function as well as the visualization of these functions along with their respective Monte-Carlo envelopes. Tests for complete spatial randomness (CSR) of point patterns based on L- and pair-correlation (L, PC) functions were performed using the Maximum Absolute Deviation (MAD) test that provided the test statistics for deviation from CSR (MADL and MADPC, respectively). The computed functional statistics provide information about spatial dependency of all points over different scales of the pattern. These test indicate the presence and the degree of clustering within a given point pattern. To test for differences between spatial point patterns of the control and diabetic groups as a whole, the studentized permutation test for L-functions and pair-correlation functions was employed and the latter were visualized along with their Monte-Carlo envelopes in the two groups. This test allows the direct comparison of point pattern groups on the basis of their functional summary characteristics without the need for extracting scalar characteristics from the functions and thereby omitting valuable spatial information. Resulting p<0.05 indicates statistically significant differences between the pooled point pattern groups (SPTL, when summary statistic is based on L-functions; SPTPC, when summary statistic is based on PC-functions).

A flow-chart of all image processing steps along with the calculated parameters is shown in S1 Fig.

**Description of SPPA**

**1. Image data preprocessing and image data preparation**

Mosaic corneal confocal microscopy (CCM) images were generated from image stacks of the corneal subbasal nerve plexus (SNP) by means of image registration, reconstruction and fusion (S2 Fig; A, B). Subsequently, corneal nerve fiber networks in these images were segmented, corrected for image artifacts or unwanted structures (such as Langerhans cells and fibrotic tissue), and interrupted nerve fiber progressions were reconstructed (S2 Fig; C, D). A detailed description of applied image (pre-)preprocessing methods has been published elsewhere (1).

The segmented nerve fiber networks were projected onto the original image area (actual acquired SNP layer content) and all nerve fibers were topologically skeletonized. Coordinates of corneal nerve branching points (CNBPs, hereafter referred to as ”points“ or ”point pattern“) in nerve fiber skeletons were determined (S2 Fig; E, F) and respective binary masks of the image area were generated. Spatial point pattern analysis (SPPA) was performed using Mathematica 10.1.0.0 (2) and spatstat 1.42-2 (3) for R 3.2.2 (4). The aim of this analysis was to determine the nature of all point patterns (clustered, random or regular, S3 Fig; A - C) and to calculate the degree of clustering. We wanted to combine different approaches for the analysis of spatial point patterns. This ranges from simple neighborhood distances over area as well as aggregation measurements up to functional statistics and direct point pattern group comparisons.

**2. Point number, density and empty space**

For each point pattern the total number of points (branching points; BP [n]) was counted and the point density (branching point density; BPD (n/total image area)) was calculated. BPD is the normalized version of BPs. This parameter refers to the number of points divided by the total image area, and thus represents a weighted measure that can be used for the comparison of images with widely differing areas. Densities were visualized using density plots in which the spatial distribution and local accumulation or absence of points in point patterns can be easily perceived (S4 Fig; A, B). Dense nerve fiber networks possess higher numbers of BPs and higher BPD, while a low number of BPs / lower BPD indicate scarce or heavily interrupted networks.

Empty space between points (empty space distance; ESD [µm]) was calculated by applying a distance transform with respect to all points. The resulting local distance image values correspond to the distance of the individual image pixels to the nearest point of the pattern. Distance image values of all pixels were averaged, providing a useful parameter for the characterization of the empty space extents within the point pattern of the images. Dense BP patterns contain narrow empty areas while sparse patterns may express vast connected empty areas, the latter indicating poorly connected nerve fiber networks. Following the parameter calculation the distance maps were visualized (S4 Fig; C, D).

**3. Nearest neighbor indices**

Nearest neighbor indices were derived from distances between neighboring points or their relations in general. They provide information about point pattern features over short distances only.

Nearest neighbor distances of the point patterns were calculated on the basis of Delaunay triangulation (5) that was corrected for border shape (S5 Fig; A - D). This triangulation method transforms a point pattern into a set of triangles. Their vertices correspond to the original points and their edges reflect connecting lines between neighboring points (S6 Fig; A).

The analyzed images exhibit irregular borders with occasional deep incisions into the image area. These are artifacts that were introduced during image reconstruction and image fusion. Since there is no information about potential further points outside the image area, it is inaccurate to calculate distances between points that are separated by concave or distorted image borders. Delaunay triangulation is applied to the point pattern under the constraint that only such connections between points are created that are not interrupted by the image background, as above mentioned incisions would do. This edge correction ensures that only actual image area is used for the calculation of Delaunay triangulation based distance measurements. The final triangulation enables easy identification of neighboring points and distance calculations on the basis of Euclidean distances. Altogether four parameters were calculated: the average minimum distance MINN (µm) and the average mean distance MENN (µm), as well as the respective average standard deviations of individual minimum (MINNSD [µm]) and mean (MENNSD [µm]) distances.

MINN is based on distances of points to their nearest neighboring points only, while the average mean distance MENN reflects the mean distance of points to all their neighboring points. Since MINN considers only the most closely related pairs of points it can be used to used gain information about the shortest distances between BPs. Dense nerve fiber networks feature more BPs which are located more closely to each other, resulting in lower MINN values. MENN, on the contrary, reflects a weighted measure that is based on all direct neighbors of a BP and is more resistant against point patterns with a higher number of spatially close pairs of points (although such patterns may not be common). MENN provides a good overview regarding the average distances of BPs. Dense nerve fiber networks show low parameter values, while networks with decreasing degrees of connectivity feature increasing MENN values.

Low values of MINNSD and MENNSD indicate minor variations of neighboring distances, thus reflecting more evenly structured nerve fiber networks. High values indicate neighborhood distance fluctuations and the presence of increasingly clustered BP patterns which may reflect a clumped network structure.

**4. Voronoi cell area**

Another measure of inter-point distances is the area of influence of points in a pattern. Voronoi tesselation (6) of point patterns was computed by partitioning the point pattern plane into non-overlapping regions, or “Voronoi cells”. For each point of the pattern there exists a corresponding surrounding cell that consists of all space that is closer to that point than to any other. The points mark the cell centers and the lines indicate borders between neighboring cells (S6 Fig; B). The Voronoi diagram of a point pattern is the dual graph to its Delaunay triangulation (S6 Fig; C).

All cells intersected by the irregular image border were adapted to the border shape (S5 Fig; E-H). This edge correction ensures that only actual image area is used for the calculation of the Voronoi cell areas. Two parameters were calculated: the mean area of all Voronoi cells of the image (VCA [µm^2^]) as well as the standard deviation (VCASD [µm^2^]).

VCA indicates how much empty area can be found around the individual BPs. The BP's cells consists of all space that is closer to their respective BP than to any other. Dense BP patterns contain a high number of small area cells while sparse patterns may express fewer cells with larger areas, the latter indicating poorly connected nerve fiber networks.

Low values of VCASD indicate minor variations of Voronoi cell areas, thus reflecting more evenly structured nerve fiber networks. High values indicate fluctuations of the Voronoi cell area sizes and the presence of increasingly clustered BP patterns which may reflect a clumped network structure.

**5. Clark-Evans aggregation index**

The spatial structure of point patterns can also be characterized by the Clark**-**Evans index (CE) of aggregation (7) in its edge-corrected version (8). This index measures the degree, to which a point pattern differs from random distribution. It is calculated using

where *N* is the total number of points, *r* represents the distance between points, *A* is the image area and *P* the length of the image border.

CE measures the degree, to which a point pattern differs from random distribution. A randomly distributed pattern (homogeneously configured nerve fiber network) shows CE=1.0, regularity of a point pattern is indicated with CE>1.0, while a clumped or clustered point pattern (clumped or clustered nerve fiber network) can be assumed when CE<1.0. With this dimensionless parameter the spatial structure of point patterns can be directly characterized and different point patterns can be compared to each other.

**6. Functional statistics**

Second order spatial analysis includes functions that are derived from point patterns and provide information about spatial dependency of all points over different scales of the pattern. Respective results can then be related to theoretical distributions (reference null-models). A common and simple reference null-model is the homogenous Poisson process (9,10) which generates point patterns of random spatial distribution, also referred to as complete spatial randomness (CSR).

A conventional function is Ripley's *K*-function (11) that provides information about spatial dependency of all points over different scales of the pattern. It is defined as

with λ as the point density (number of points per image area). The unbiased estimator of the *K*-function is

where *n* is the number of points in the image area *A*, *u_ij_* represents the distance between the *i*-th and the *j*-th point, *I_r_(u_ij_)* is an indicator function which equals 1 when *u_ij_*≤*r* and 0 otherwise, and *w_ij_* is the edge correction factor (12,13). In principle, a circle of radius *r* is constructed around each point *i*, the number of other points *j* within this circle is counted (S7 Fig; A) and the proportion of the circle that lies within the image area is calculated. These three steps are repeated for all points *i* and the result is accumulated. Then, *r* is being incremented by a small amount and the procedure is repeated.

Under the assumption of CSR the expected number of points within the distance *r* of a point is

*K(r)* is usually plotted against *r* and departure from CSR can be observed when *K(r)*<*πr^2^* (indicating a regular pattern) or *K(r)*>*πr^2^* (indicating a clustered pattern).

Interpretation of the K-function is simplified when it is transformed into its standardized, variance-stabilized version *L(r)* (14):

Under the assumption of CSR the expected number of points within the distance *r* of a point is

*L*(*r*)=0

*L(r)* can be plotted against r and departure from CSR can be observed when *L(r)*<0 (indicating a regular pattern) or *L(r)*>*0* (indicating a clustered pattern) (S8 Fig; A, B; green lines).

While *K*-function and *L*-function are of cumulative character and measure the number of points up to a defined distance, the *pair-correlation* function *g(r)* (15):

is non-cumulative and provides information about regularity or clustering at a particular distance. The procedure resembles the calculation of the *K*-function, with the difference that only those points are counted that are located within a narrow annulus of diameter *d* at a circle of radius *r* around each point (S7 Fig; B). CSR is indicated when *g(r)*=1 holds true, while regularity is suggested when *g(r)*<1, and clustering when *g(r)*>1 (S8 Fig; C, D; green lines). For all point patterns *L(r)* and *g(r)*-functions were calculated.

**7. Test for** **complete spatial randomness**

Point patterns can be tested for departure from complete spatial randomness (CSR), whereby CSR acts as a ”dividing line“ between regular and clustered patterns (16). Such tests can be categorized into envelope and deviation tests (17).

Envelope tests (11) compare the observed summary characteristic from a function (e.g. the *g*-function) of a given point pattern to estimates, obtained from a simulation of the null-model, which uses the estimated parameters in the same image area. Generally, the null-model is simulated a number of times, and the extreme values from all simulations *g_min_(r)* and *g_max_(r)* of estimator *ĝ(r)* are determined. Extreme values can be plotted together with the estimator *ĝ(r)* and form the envelopes of *ĝ(r)* (S8 Fig; light green areas). If *ĝ(r)* is located outside the envelope at a certain distance *r*, the null hypothesis of CSR is rejected for that distance *r*. The "direction" of significant departure from CSR is indicated by the position of the function value relative to the envelope: below envelope hints at regularity while above envelope suggests clustering (S8 Fig; A - D). For each *r*, and in conjunction with the respective point pattern, it could then be discussed why the associated function value is located outside the calculated envelope.

Deviation tests calculate the deviation between the observed function and the expectation under the null hypothesis. Hereby, the information of the functional summary statistic is transformed into a scalar test statistic. After calculation of the deviation measure the p-value of the deviation test can be estimated. The maximum absolute deviation (MAD) test (11,18) was used to test point patterns for CSR. This tests calculates a scalar test statistic (MADL, when based on *L(r)*-functions; MADPC, when based on *g(r)*-functions) and thus provides a measure for how much a given point pattern departs from CSR. After calculation of the deviation measure the p-value of the deviation test can be estimated. A p-value <0.05 indicates point patterns that differ significantly from CSR. The percentage of point patterns that depart from CSR can then be reported as a separate parameter (MADPL (%), when based on L-functions; MADPPC (%), when based on PC-functions). The degree of departure from CSR can be interpreted as a measure for the clustering of a given point pattern. Very dense fiber networks typically show no departure from CSR as their BPs are randomly positioned. Increasingly sparse or damaged nerve fiber networks show a slight departure from CSR indicating a clumping pattern of BPs on different scales, while rudimentary or heavily damaged networks show heavy clumping or only a few remaining BPs.

**8. Test for differences between groups of point patterns**

*L(r)* and *g(r)* functions from control and diabetic group were pooled and tested for group differences using the studentized permutation test (19). This test allows the direct comparison of point pattern groups on the basis of their functional summary characteristics without the need for extracting scalar characteristics from the functions and thereby omitting valuable spatial information. Resulting p-values <0.05 indicate statistically significant differences between the pooled point pattern groups (SPTL, when summary statistic is based on *L(r)*-functions; SPTPC, when summary statistic is based on *g(r)*-functions).

**S1 Fig. Flow-chart of all image processing steps along with the parameters calculated.**


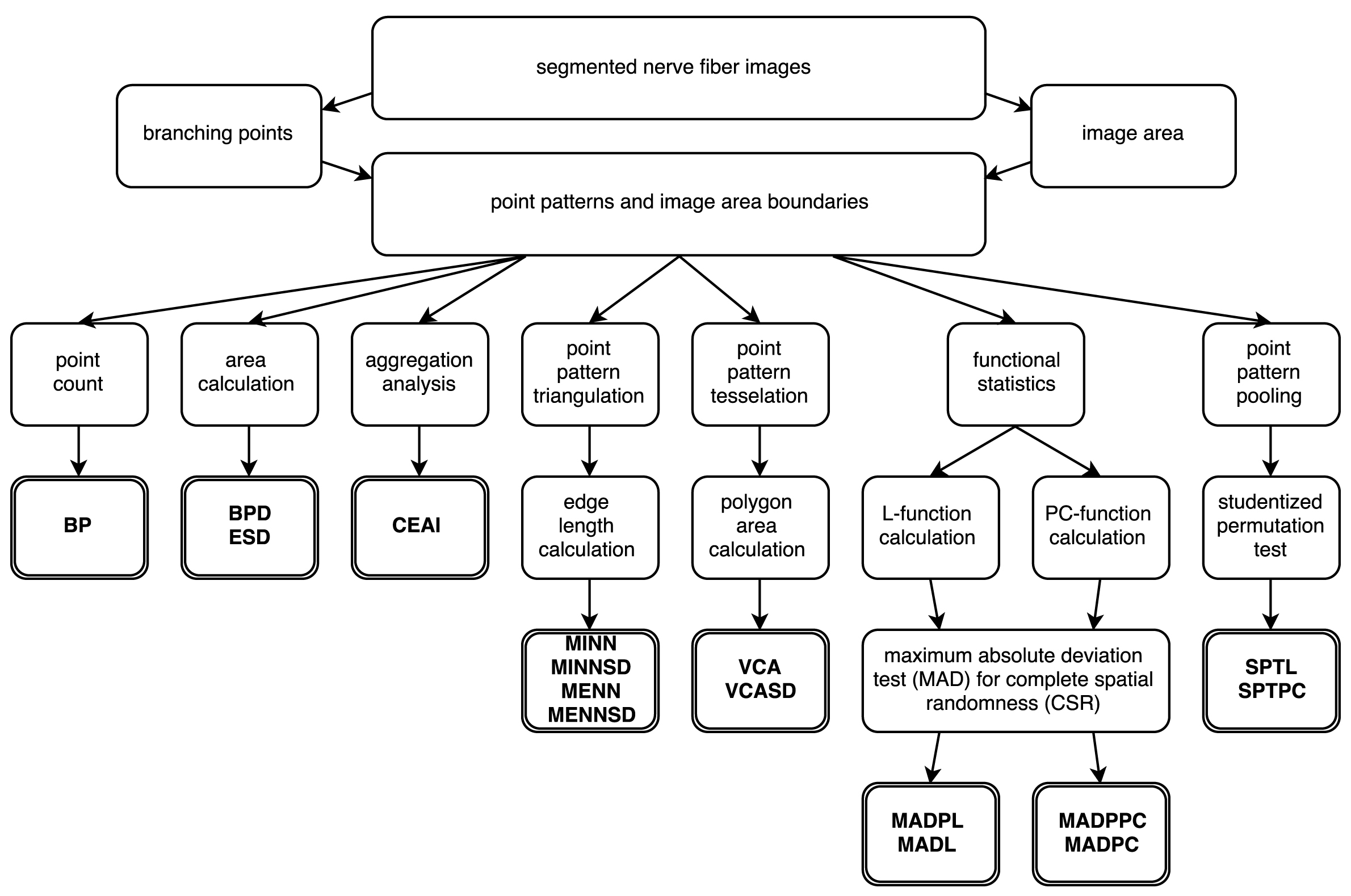


**S2 Fig. Example CCM images from control (A) and diabetic (B) group, respective segmented nerve fiber networks (C, D) and related branching points (E, F).** The white frame shows the size of an individual HRT image (400x400 µm) for comparison.


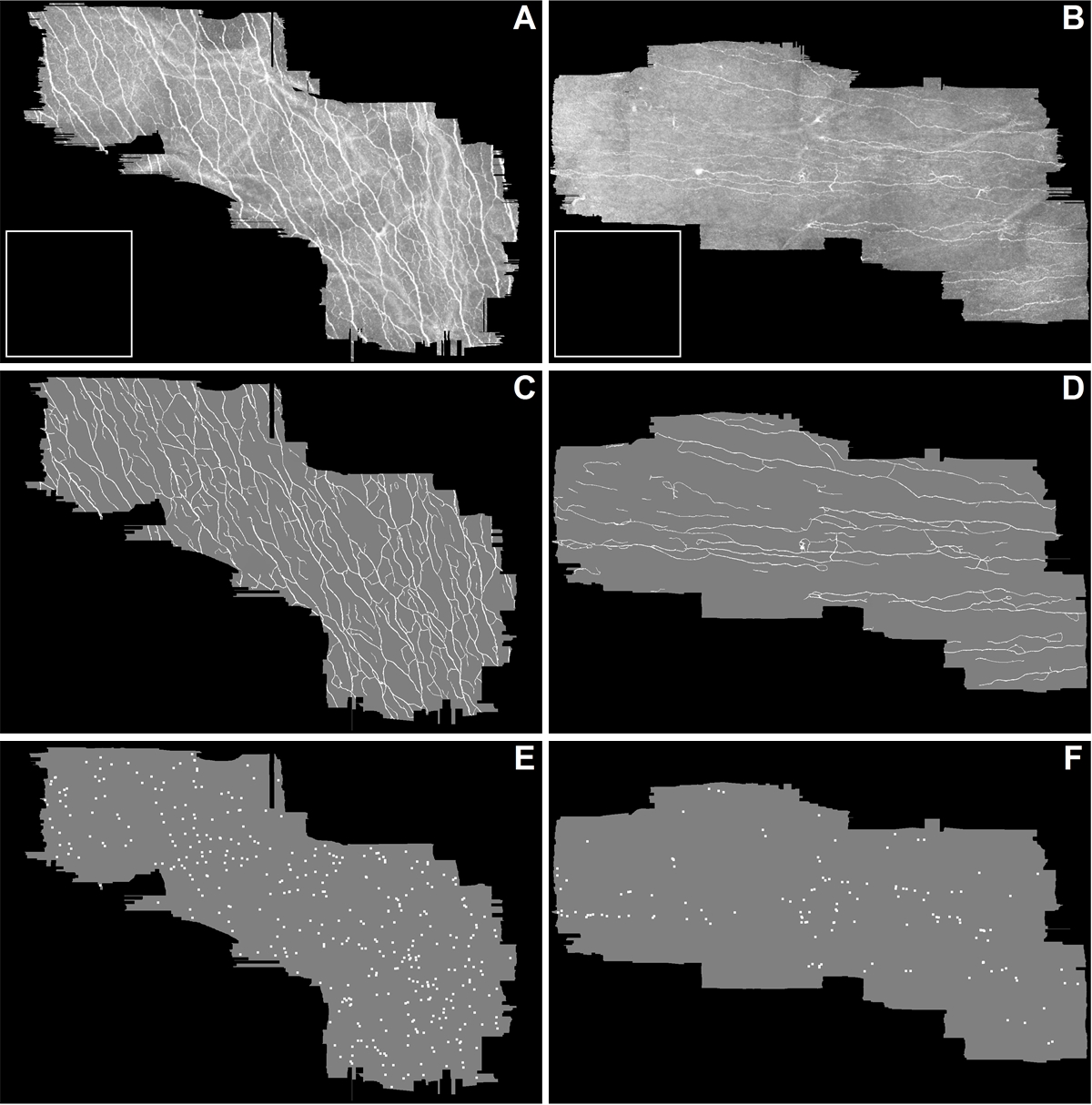


**S3 Fig. Three fundamental types of point patterns: clustered (A), random (B) and regular (C).**


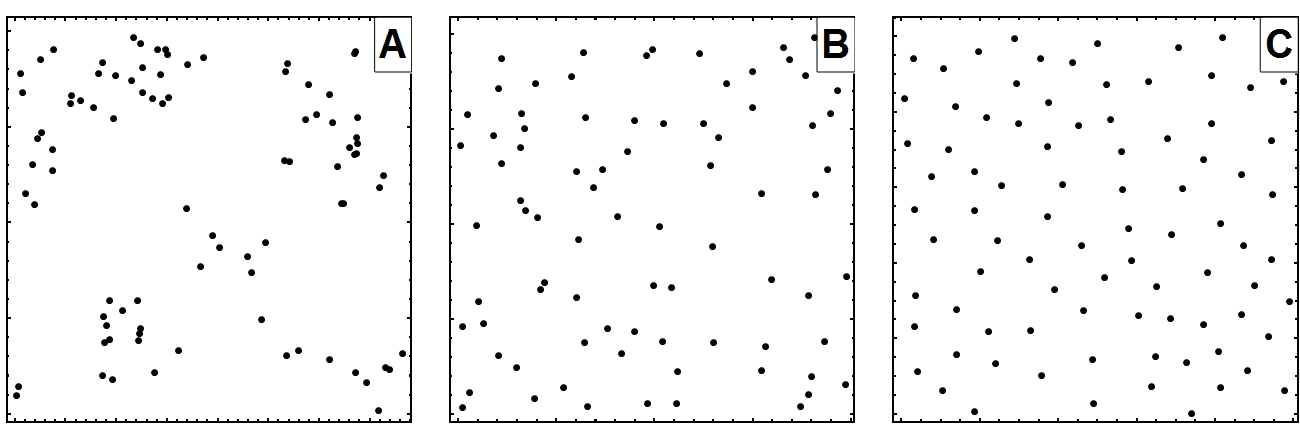


**S4 Fig. Density maps (A, B) and empty space plots (C, D) for example images from control (left column) and diabetic (right column) group.**


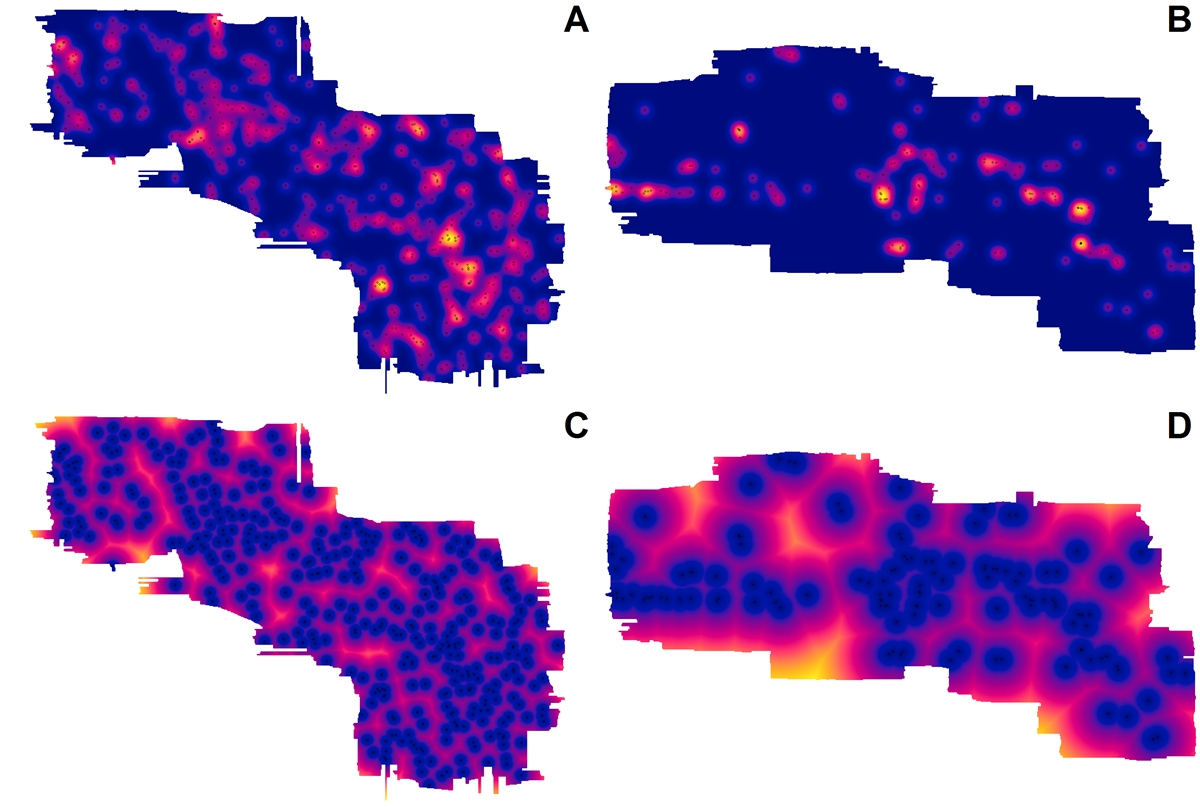


**S5 Fig. Delaunay triangulations (A, B; edge corrected: C, D) and Voronoi tesselations (E, F; edge corrected: G, H) for example images from a control subject (left column) and diabetic patient (right column).**


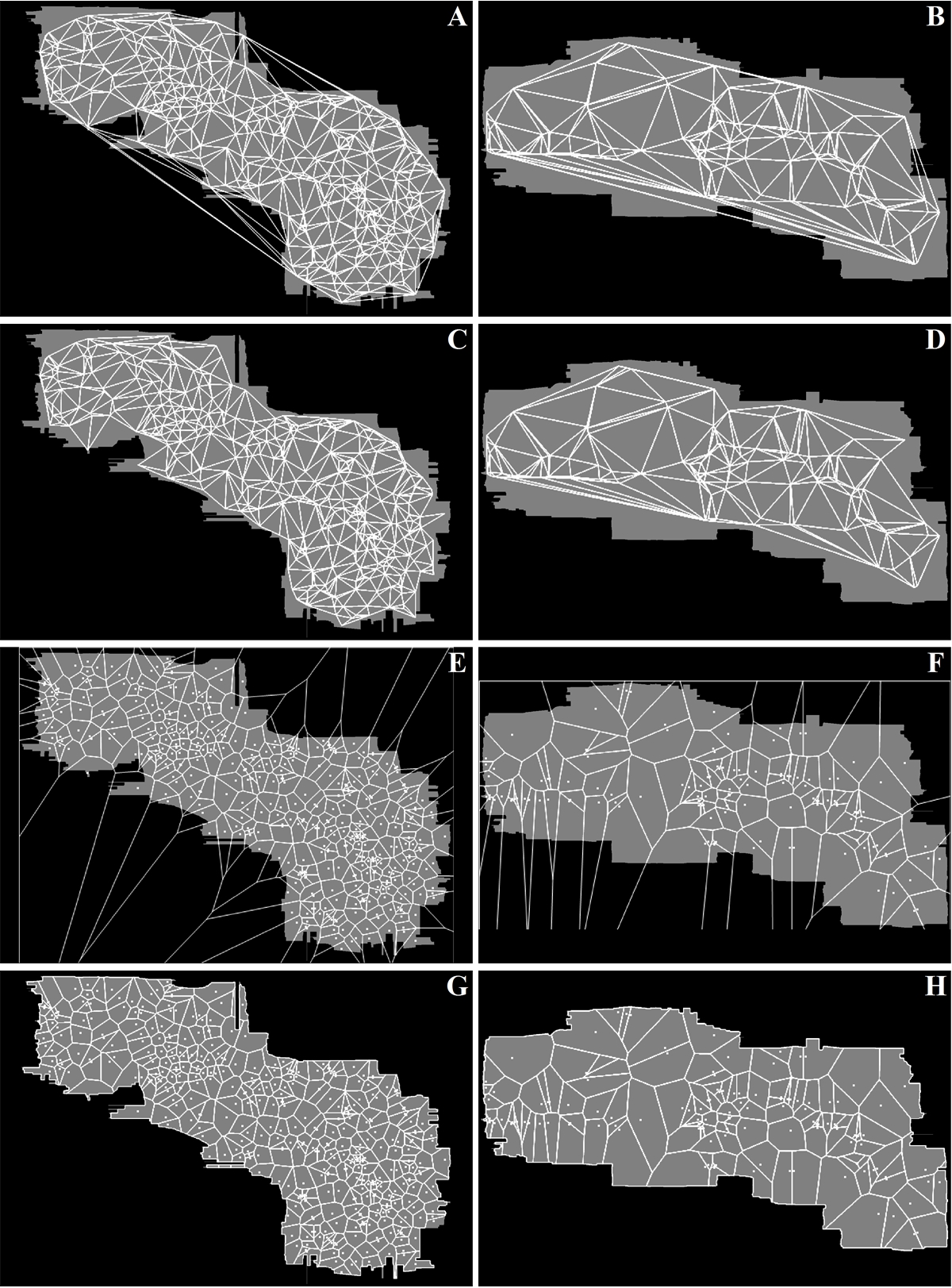


**S6 Fig. Delaunay triangulation (A) and Voronoi tesselation (B) for a set of points along with a combined illustration (C).**


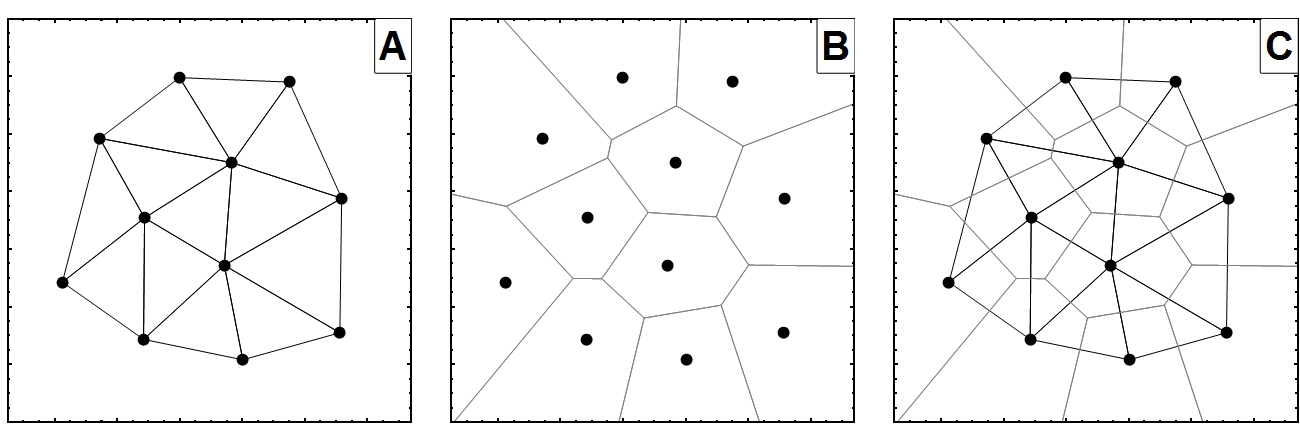


**S7 Fig. Functional statistics.** Subsets of relevant points (black) for the calculation of *L(r)* and *g(r)*-functions of a given radius: Starting from a chosen point the *L(r)*-function is calculated from all points that are located within a continuously growing circle of radius *r* around the respective point (A), while the *g(r)*-function is calculated from all points that are located within a narrow annulus of diameter *d* at a continuously growing circle of radius *r* around the respective point (B). *L(r)* and *g(r)*-functions of a given point pattern at a radius *r* are calculated for all points simultaneously.


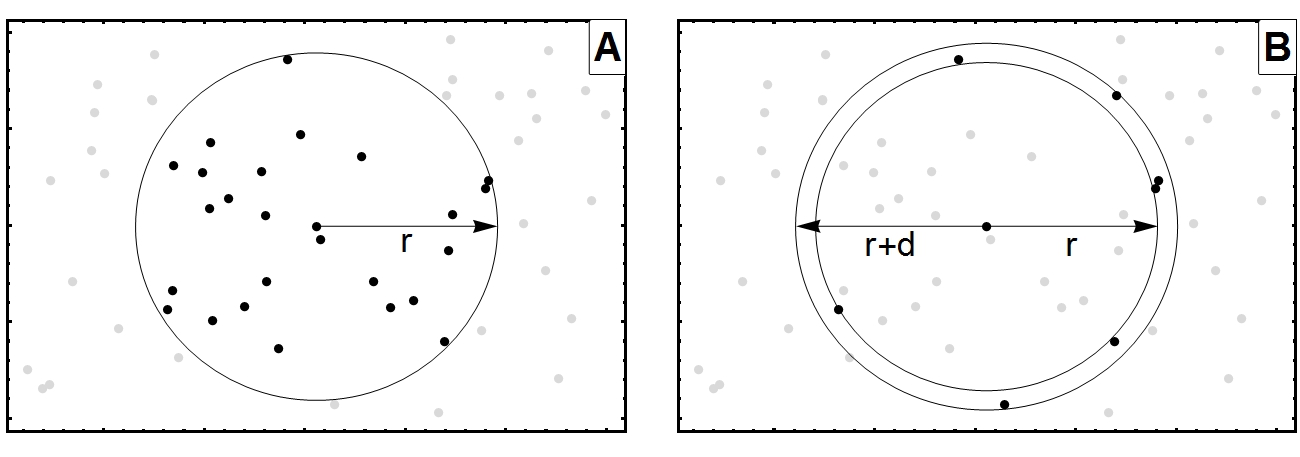


**S8 Fig. Test for complete spatial randomness.** *L(r)* (A, C) and *g(r)* (B, D) for example images from control (left column) and diabetic (right column) group. Respective point patterns were taken from Fig. 1 (E, F). Plots show functions (green lines), computed envelopes (light green areas) and assumption of CSR (black lines) for the respective functions. Functions leaving the envelope indicate significant departure from CSR at the respective distance *r* (below envelope = regularity, above envelope = clustering).


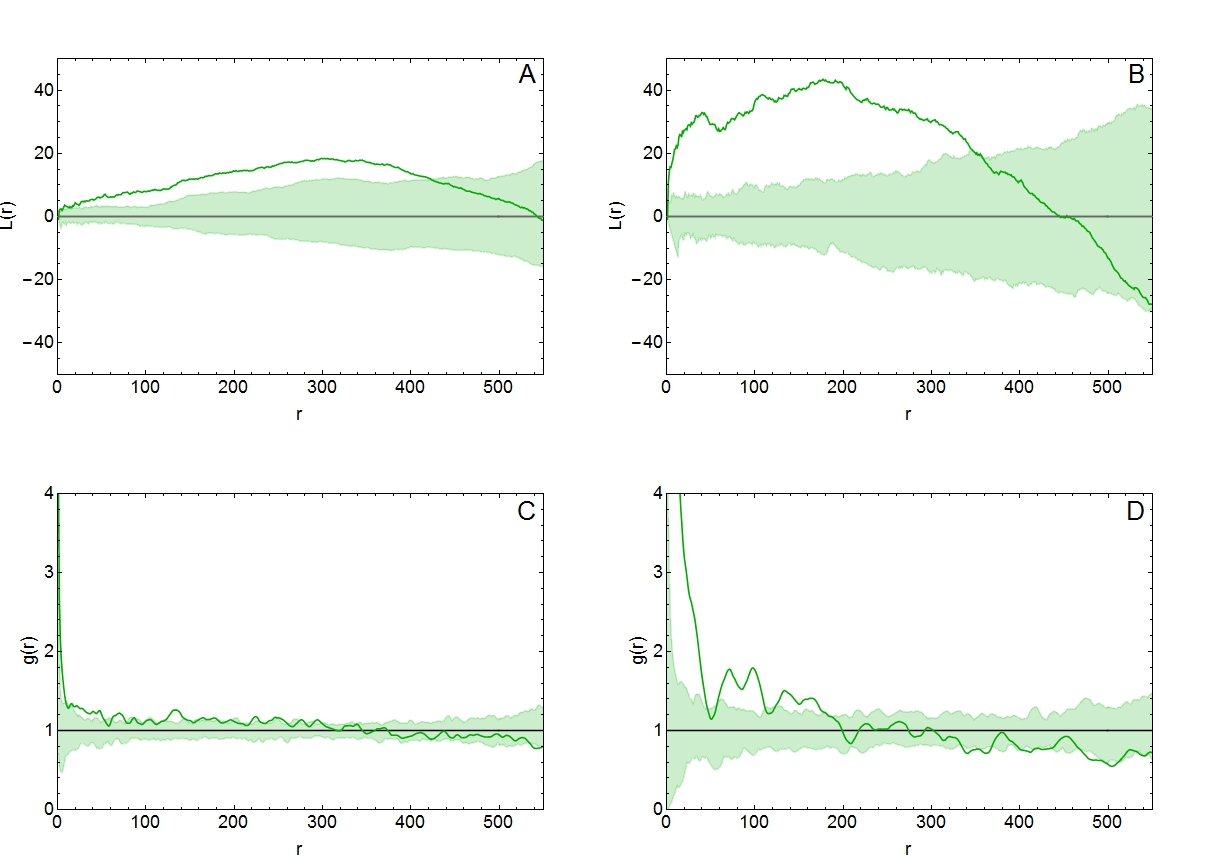


**References**

1. Ziegler D, Papanas N, Zhivov A, Allgeier S, Winter K, Ziegler I, Brüggemann J, Strom A, Peschel S, Köhler B, Stachs O, Guthoff RF, Roden M, for the GDS Group. Early detection of nerve fiber loss by corneal confocal microscopy and skin biopsy in recently diagnosed type 2 diabetes. Diabetes 2014; 63: 2454-2463.
2. Wolfram Research, Inc., Champaign, IL, 2015.
3. Baddeley A, Turner R. Spatstat: An R Package for Analyzing Spatial Point Patterns. Journal of Statistical Software 2005; 12: 1-42.
4. R Core Team. R. A language and environment for statistical computing. R Foundation for Statistical Computing, Vienna, Austria, 2015.
5. Delaunay, Boris N. Sur la sphère vide. Bulletin of Academy of Sciences of the USSR 7 1934; 6: 793-800.
6. Voronoi, G. Nouvelles applications des paramètres continus à la théorie des formes quadratiques. J Reine Angew Math 1907; 133: 97-178.
7. Clark PJ, Evans FC. Distance to nearest neighbor as a measure of spatial relationships in populations. Ecology 1954; 35: 445–453.
8. Sinclair DF. On tests of spatial randomness using mean nearest neighbour distance. Ecology 1985; 66:1084–1085.
9. Illian J, Penttinen A, Stoyan H, Stoyan D. Statistical Analysis and Modelling of Spatial Point Patterns. Wiley, Chichester, 2008.
10. Cressie NAC. Statistics for spatial data. 2^nd^ edition Wiley, New York, 1993.
11. Ripley BD. The second-order analysis of stationary point processes. J Appl Probability 1976; 13: 255-266.
12. Szwagrzyk J. Small scale spatial patterns of trees in mixed pine-beech forests. Forest Ecology and Management 1992; 51: 301-315.
13. Haase P. Spatial pattern analysis in ecology based on Ripley’s K-function: introduction and methods of edge correction. Journal of Vegetation Science 1995; 6: 575–582.
14. Besag J. Discussion of Dr Ripley's paper. J Royal Stat Soc, Series B, 1977; 39: 193–195.
15. Stoyan D, Stoyan H. Fractals, random shapes and point fields: Methods of geometrical statistics. 1994 Chichester, Wiley.
16. Diggle PJ. Statistical Analysis of Spatial and Spatio-Temporal Point Patterns, 2003 2nd Edition, Chapman and Hall/CRC.
17. Szmyt J. Spatial statistics in ecological analysis: From indices to functions. Silva Fennica. 2014; 48: article id 1008.
18. Ripley BD. Modelling spatial patterns (with discussion). J Royal Stat Soc, Series B, 1977; 39: 172–212.
19. Hahn, U. A studentized permutation test for the comparison of spatial point patterns. Journal of the American Statistical Association 2012; 107: 754-764.
